# Comparison of the Efficacy of Pregabalin and Gabapentin for Preemptive Analgesia in Laparoscopic Cholecystectomy Patients: A Randomised Double-Blind Study

**DOI:** 10.7759/cureus.46719

**Published:** 2023-10-09

**Authors:** Simrit Kaur, Sartaj Turka, Tripat Kaur Bindra, Rajan D Tuteja, Manoj Kumar, Sukhminder Jit Singh Bajwa, Madhuri S Kurdi, Apoorva J Sutagatti

**Affiliations:** 1 Department of Anaesthesia and Intensive Care, Government Medical College (GMC) Patiala, Patiala, IND; 2 Department of Medicine and Surgery, Government Medical College (GMC) Patiala, Patiala, IND; 3 Department of Anaesthesia, Anugrah Narayan Magadh Medical College and Hospital, Gaya, IND; 4 Department of Anaesthesiology and Intensive Care, Gian Sagar Hospital & Medical College, Rajpura, IND; 5 Department of Anaesthesiology, Karnataka Institute of Medical Sciences, Hubli, IND; 6 Department of Medicine and Surgery, S. Nijalingappa Medical College and H.S.K. Hospital and Research Centre, Bagalkot, IND

**Keywords:** preemptive, tramadol, sedation, postoperative, pregabalin, pain, laparoscopic cholecystectomy, gabapentin

## Abstract

Introduction

Preemptive analgesia is now an essential step of perioperative pain management. Pregabalin and gabapentin, which are drugs primarily used in the treatment of neuropathic pain, are now being contemplated for use as preemptive analgesics. This study aimed to assess the effectiveness of gabapentin and pregabalin as preemptive analgesics. The primary objective of the study was to compare pregabalin and gabapentin versus placebo with regard to a visual analogue scale (VAS) score for postoperative pain for 24 hours, time to first rescue analgesia, and total analgesic consumption over 24 hours. The level of sedation with the help of a modified Ramsay sedation score was also compared.

Methods

This randomised, double-blind study was conducted on 90 patients aged 18-60 years of the American Society of Anesthesiologists (ASA) physical status I and II undergoing elective laparoscopic cholecystectomy under general anaesthesia at a tertiary health care institute. The patients were randomly divided into three groups of 30 each, namely, Group A (gabapentin - oral two capsules of 300 mg gabapentin), Group B (pregabalin - oral two capsules of 150 mg pregabalin), and Group C (placebo - oral two capsules). The various parameters that were recorded in both groups included a VAS score for pain, total dose of tramadol consumed in 24 hours, modified Ramsay sedation scores in the immediate postoperative period, and adverse effects related to the study drugs (at zero and one hour and two, four, six, 12, and 24 hours). The data were analysed using the Statistical Package for the Social Sciences (SPSS) (version 25; IBM SPSS Statistics for Windows, Armonk, NY) software.

Results

VAS scores were significantly lower in groups A and B when compared to Group C. However, the scores were comparable in Group A (gabapentin) and Group B (pregabalin). The difference in the mean time of rescue analgesia was statistically highly significant when Group A (gabapentin) was compared with Group C (placebo) (P value<0.001) and when Group B (pregabalin) was compared with Group C (placebo) (P value<0.001). Thus, gabapentin and pregabalin provide a longer postoperative pain-free period (382.6 min and 502.3 min, respectively) when compared to the placebo group (137.8 min). Moreover, the mean dose of tramadol consumption in 24 hours was significantly lower in pregabalin (170 mg) and gabapentin groups (176.7 mg) when compared to the placebo group (286.7 mg). However, there was no significant difference in the total tramadol consumption between the gabapentin and pregabalin groups. The level of sedation up to six hours postoperatively was higher in Group B (pregabalin) and Group A (gabapentin) patients compared to Group C (placebo). On comparing the mean Ramsay sedation scores of Group A (gabapentin) versus Group C (placebo) and Group B (pregabalin) versus Group C (placebo), it was found that there was a highly significant difference at zero and one-hour time intervals (P value<0.001 in both comparisons).

Conclusion

A single preoperative dose of pregabalin 300 mg or gabapentin 600 mg can be used for effective preemptive analgesia in patients undergoing laparoscopic cholecystectomy.

## Introduction

The incidence of postoperative pain is around 80% [1]. Surgical trauma-induced hyperalgesia can lead to chronic pain in the postoperative period [2]. Inadequately treated postoperative pain may have various systemic implications such as tachycardia, hypertension, increased blood glucose, delayed wound healing, and anxiety. Preemptive analgesia is an intervention done before a surgical incision is taken. This produces better postoperative analgesia and facilitates early mobilisation and functional rehabilitation after surgery. Preemptive analgesia focuses on reducing postoperative opioid consumption and pain levels, decreasing the incidence of adverse events, and improving patient satisfaction. Several preemptive analgesic regimens have been tried in the perioperative period, including opioids, nonsteroidal anti-inflammatory drugs, and so on. Pregabalin and gabapentin are amino acid derivatives of gamma-aminobutyric acid (GABA) and are currently emerging drugs in this field. Both gabapentin and pregabalin share similar mechanisms in preventing postoperative pain. Their postsynaptic binding to the alpha2-delta subunit of the dorsal horn neurons’ voltage-dependent calcium channels causes a decrease in the entry of calcium into the nerve endings and thus decreases the release of neurotransmitters such as noradrenaline. Although their use for the management of postoperative pain is off-label, their perioperative oral use has become widespread. The efficacy of perioperative pregabalin or gabapentin administration in the prevention of acute postoperative pain has been studied by various authors [3].

There is literature suggesting that gabapentin and pregabalin produce prolonged postoperative analgesia compared to placebo and that pregabalin has a better analgesic profile in the postoperative period with lower a visual analogue scale (VAS) score, prolonged timing of first rescue analgesic, and less opioid consumption than gabapentin. Patients receiving gabapentinoids have lower postoperative VAS scores, prolonged timing of first rescue analgesia, and less opioid consumption [4-6].

Laparoscopic cholecystectomy is a commonly conducted surgical procedure. Thus, this study was conducted to assess the effectiveness of gabapentin and pregabalin as preemptive analgesics in patients undergoing laparoscopic cholecystectomy. The primary objective was to assess and compare gabapentin and pregabalin versus placebo with regard to VAS score for postoperative pain for 24 hours, the time to first rescue analgesia, and total analgesic (tramadol) consumption over 24 hours. The level of sedation with the study drugs was also compared with the help of a modified Ramsay sedation score [7]. The secondary objective of the study was to look for side effects related to the study drugs.

## Materials and methods

**Figure 1:**
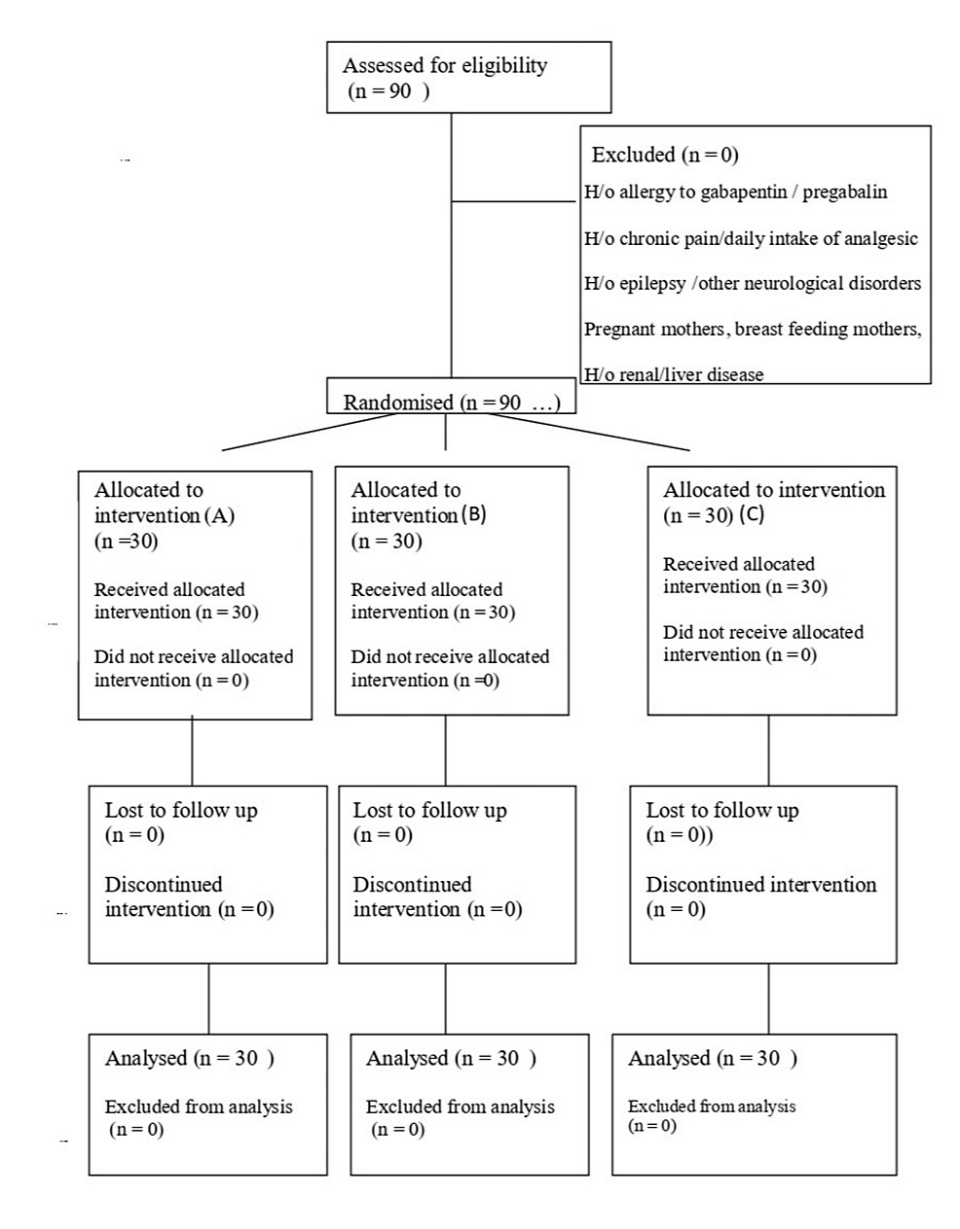
Consolidated Standards for the Reporting of Trials (CONSORT) diagram H/o: History of; n: Number

This randomised, double-blind study was conducted on 90 patients (Figure 1) aged 18-60 years, of the American Society of Anesthesiologists (ASA) physical status I and II undergoing elective laparoscopic cholecystectomy under general anaesthesia at a tertiary medical college hospital after obtaining permission from the Institutional Ethics Committee of Rajindra Hospital, Government Medical College, Patiala (BFUHS/2K21p-TH/14755 dated 15/12/21). The study was conducted for a period of nine months from January 2022 to September 2022 in accordance with the principles of the Declaration of Helsinki of 1975 as amended in 2013. Patients of either gender were included in the study. Patients with a history of allergy to gabapentin and pregabalin, patients who were prescribed pregabalin or gabapentin for other indications, patients having a history of chronic pain and chronic daily intake of analgesics, those with a history of epilepsy and other neurological disorders, pregnant women, breastfeeding mothers, and patients with liver or renal disease were excluded from the study. A simple randomisation technique using computerised random numbers was followed to assign either of the three groups to the patient (Group A/B/C). Group allocation was concealed by using serially numbered, sealed, and opaque envelopes. The drug/placebo was administered by an anaesthesiologist who was aware of the group that the patient belonged to. However, the patients participating in the study were unaware of the drug that was administered, and the anaesthesiologist who gathered the postoperative data was also not aware of the group that the patient belonged to.

The sample size was estimated based on a previous study [4], wherein pregabalin was compared with gabapentin for postoperative analgesia in abdominal hysterectomy cases. Based on this study, the anticipated consumption of tramadol was 42.78 mg (standard deviation=18.73 mg) in the pregabalin group and 54.40 mg (standard deviation=15.57 mg) in the gabapentin group, and a reduction in the dose in pregabalin group by 20% was considered significant. The sample size was calculated assuming 90% power and 95% confidence interval using the formula: n=(r+1)/r SD2 (Zβ- Zα)2/(d) 2, where r=1; (r+1)/r=2; SD=0.1; Zβ=0.90; Zα = 1.96; d=0.25 (n: sample size; SD: standard deviation; d: effect size; r: ratio of control to cases; Zβ: the desired type II error; Zα: level of statistical significance). A minimum of 26.574=27 patients were required for each group in the study. We took a sample size of 30 in each group to adjust for dropouts.

Preanaesthetic checkups and investigations were done for every patient before the surgery. Informed written consent was obtained from the patient after providing complete details regarding the study protocol and the procedure. After preanaesthetic evaluation and basic laboratory investigations, the patient was explained to in detail about the procedure during the preanaesthetic visit. The patient was familiarised with the VAS for pain on the day before surgery. Group A (gabapentin) - the patient was given oral two capsules of 300 mg gabapentin (total 600 mg) with sips of water one hour before the induction of anaesthesia. Group B (pregabalin) - the patient was given oral two capsules of 150 mg pregabalin (total 300 mg) with sips of water one hour before the induction of anaesthesia. Group C (placebo) - the patient was given oral two capsules of a matching placebo (capsule containing powdered multivitamin) with sips of water one hour before the induction of anaesthesia.

In the operation theatre, intravenous access was obtained and a fluid infusion was started. The baseline values of heart rate, peripheral oxygen saturation (SpO2), mean blood pressure, and respiratory rate were recorded. This was followed by the intravenous injection of glycopyrrolate 0.2 mg and fentanyl 2 µg/kg.

After preoxygenation with 100% oxygen for three minutes, the patient was induced with an injection of propofol 2 mg/kg intravenously slowly, and intubation was facilitated with an injection of vecuronium 0.1 mg/kg intravenously. Anaesthesia was maintained with 33% oxygen, 66% nitrous oxide, isoflurane (0.6-1% titrated according to blood pressure (BP) readings), and vecuronium 0.02 mg/kg. The vital parameters including heart rate, systolic BP, diastolic BP, mean arterial pressure, SpO2, and end-tidal carbon dioxide (EtCO2) levels were monitored continuously and recorded every 10 minutes. Intravenous paracetamol 1 gm was administered towards the end of the surgery, and the patient was reversed with intravenous neostigmine 0.05 mg/kg and glycopyrrolate 0.01 mg/kg. The patient was extubated when fully awake. After extubation, the patient was shifted to the recovery room when fully awake, and this time was considered zero hours. VAS scores were assessed in the immediate postoperative period (zero hours) and at one, two, four, six, 12, and 24 hours. Patients were given rescue analgesic tramadol 1.5 mg/kg intravenously when the VAS score was four or greater. Sedation was assessed postoperatively by the Ramsay sedation scale at zero, one, two, four, six, 12, and 24 hours.

We looked for side effects of the study drugs such as nausea, vomiting, headache, dizziness, and respiratory depression (respiratory rate < 10 breaths/minute) in the study of patients up to 24 hours postoperatively. The data were analysed using Statistical Package for Social Sciences software (SPSS) (version 25; IBM SPSS Statistics for Windows, Armonk, NY) and the Microsoft Excel program. Descriptive statistics was used for all data, and these were reported in terms of mean, standard deviation, and percentages. Appropriate statistical tests of comparison were applied. Categorical variables like age and gender were analysed with the help of the chi-square test. A P value of < 0.05 was considered statistically significant, and a P value of < 0.001 was considered highly significant.

## Results

The distribution of patients according to age, gender, and weight was similar in both groups. Both groups were comparable and statistically nonsignificant (P value>0.05) (Table 1).

**Table 1:** Demographic parameters n: number of patients; SD: standard deviation

Demographic Parameter	Group A (n)	Group B (n)		Group C (n)			P value (chi-square)	
Gender								
Male	7	5		5			0.82	
Female	23	25		25				
Age (years)								
Mean ± SD	37.47 ± 10.228		37.00 ± 8.499		35.90 ± 7.586			0.76
Weight (kg)								
Mean ± SD	67.933 ± 8.443		68.433 ± 5.975			67.666 ± 6.854		0.73

**Figure 2:**
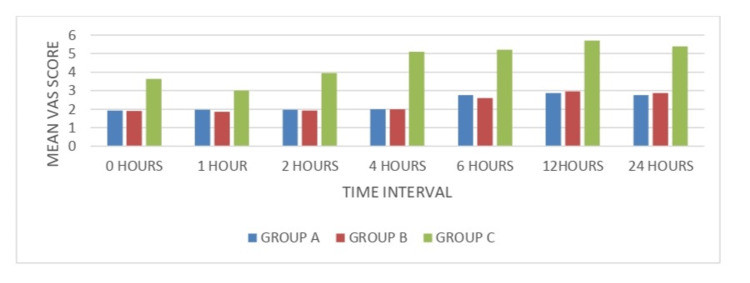
Mean visual analogue scale (VAS) score at different time intervals VAS: visual analogue scale; Group A: gabapentin; Group B: pregabalin; Group C: placebo

The mean VAS scores for pain (Table 2, Figure 2) during the postoperative period at zero, one, two, four, six, 12, and 24 hours were significantly less in Groups A (gabapentin) and B (pregabalin) when compared to Group C (placebo). However, the scores were comparable in Groups A and Group B. This means that, clinically, both gabapentin and pregabalin produce postoperative analgesia, and their analgesic efficacy is similar.

**Table 2:** Mean visual analogue scale score (for pain) at different time intervals VAS: visual analogue scale; SD: standard deviation; vs: versus; HS: highly significant; S: significant

VAS score	Group A		Group B		Group C		P value	P value	P value
	Mean	SD±	Mean	SD±	Mean	SD±	Group A vs C	Group B vs C	Group A vs B
0 h	1.93	0.18	1.91	0.25	3.64	0.91	<0.001 (HS)	<0.001 (HS)	0.21 (NS)
1 h	1.97	0.18	1.86	0.34	3.0	0.91	<0.001 (HS)	<0.001 (HS)	0.16 (NS)
2 h	1.97	0.18	1.93	0.25	3.96	0.92	<0.001 (HS)	<0.001 (HS)	0.56 (NS)
4 h	2.0	0.00	2.0	0.26	5.1	1.09	<0.001 (HS)	<0.001 (HS)	1.0 (NS)
6 h	2.76	1.10	2.6	0.77	5.2	0.37	0.007 (S)	0.015 (S)	0.50 (NS)
12 h	2.87	0.93	2.96	1.09	5.7	0.55	0.015 (S)	0.001 (S)	0.063 (NS)
24 h	2.76	1.02	2.86	0.94	5.4	1.19	0.018 (S)	0.038 (S)	0.362 (NS)

The time interval for the first dose of the rescue analgesic (tramadol) post-surgery was 382.6 minutes in Group A (gabapentin), 502.3 minutes in Group B (pregabalin), and 137.8 minutes in Group C (placebo). The difference in the mean time of rescue analgesia was statistically highly significant when Group A was compared with Group C and when Group B was compared with Group C ( P value of Group A versus C<0.001; Group B versus C<0.001). The P value for Group A versus B was significant viz 0.014 (Table 3).

**Table 3:** Mean time of rescue analgesia (in min) SD: standard deviation

Duration (in minutes)	Group A	Group B	Group C
Mean	382.6	502.3	137.8
SD	119.16	101.08	44.48
Range	220-570	220-550	60-205

The mean dose of rescue analgesic (tramadol) required in the 24 hours after surgery was 176.7 mg in Group A (gabapentin). In Group B (pregabalin), the dose required was 170 mg, while in Group C (placebo) patients, the mean dose of tramadol required was 286.7 mg. The P value was found to be <0.001 for Group A versus C and for Group B versus C, which is highly significant. The P value was 0.63 for Group A versus Group B, and this was not significant (Table 4).

**Table 4:** Mean of total dose of tramadol administered in 24 hours after surgery SD: standard deviation

Dose (in mg)	Group A	Group B	Group C
Mean	176.7	170	286.7
SD	50.40	46.60	34.57
Range	100-200	100-300	100-300

**Figure 3:**
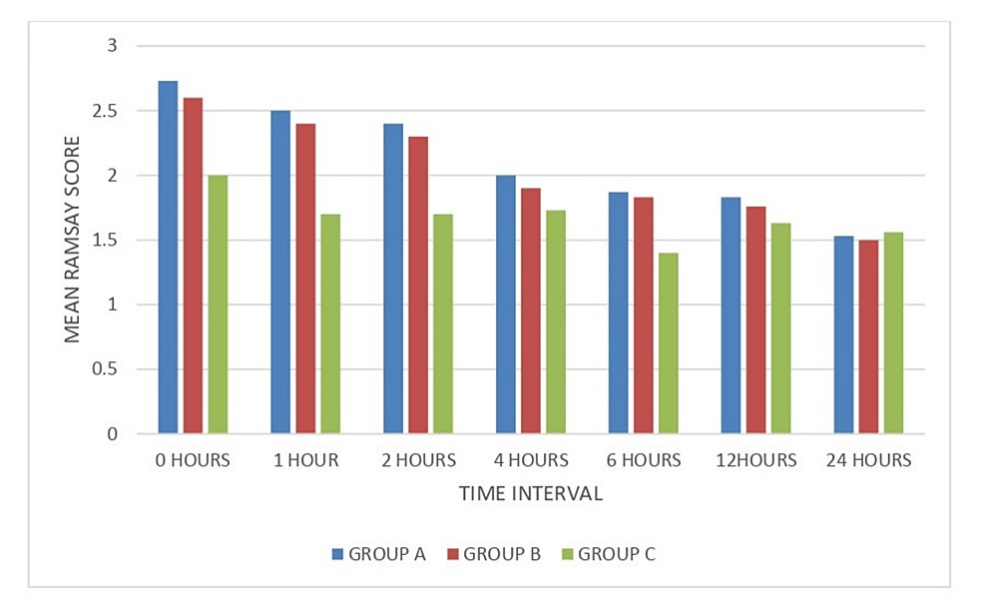
Mean modified Ramsay sedation scores at different time intervals Group A: gabapentin; Group B: pregabalin; Group C: placebo

On comparing the mean Ramsay sedation scores of Group A (gabapentin) and Group C (placebo), it was found that there was a highly significant difference at zero and one hour (P value<0.001). The difference was statistically significant afterwards until the six-hour interval (P value<0.005), after which it was statistically nonsignificant at 12- and 24-hour time intervals (P value>0.005). On comparing Group B (pregabalin) and Group C (placebo), there was a statistically highly significant difference in the mean Ramsay sedation score at zero- and one-hour time intervals (P value<0.001). However, the difference was statistically significant afterwards until the six-hour time interval (P value<0.005), after which it was statistically nonsignificant at the 12- and 24-hour time intervals (P value>0.005) (Table 5, Figure 3).

**Table 5:** Mean modified Ramsay sedation scores at different time intervals Vs: versus; HS: highly significant; NS: not significant; S: significant; SD: standard deviation

Ramsay sedation score	Group A		Group B		Group C		P value	P value	P value
	Mean	SD±	Mean	SD±	Mean	SD±	Group A vs C	Group B vs C	Group A vs B
0 h	2.73	0.407	2.60	0.31	2.0	0	<0.001 (HS)	<0.001 (HS)	0.261 (NS)
1 h	2.5	0.498	2.4	0.123	1.7	0.466	<0.001 (HS)	<0.001 (HS)	0.352 (NS)
2 h	2.4	0.305	2.3	0.325	1.7	0.466	0.002 (S)	<0.001 (HS)	0.078 (NS)
4 h	2	0	1.9	0.305	1.73	0.45	0.013 (S)	0.015 (S)	0.73 (NS)
6 h	1.87	0.183	1.83	0.490	1.40	0.498	0.016 (S)	0.019 (S)	0.786 (NS)
12h	1.83	0.592	1.76	0.430	1.63	0.490	0.159 (NS)	0.267 (NS)	0.831 (NS)
24 h	1.53	0.681	1.50	0.509	1.56	0.254	0.16 (NS)	0.345 (NS)	0.405 (NS)

Two patients each in the gabapentin and pregabalin groups complained of dizziness preoperatively and two patients in the gabapentin group had postoperative vomiting at two hours. Respiratory depression was not observed in any case. There was no significant difference in the occurrence of side effects related to the study drugs.

## Discussion

The mean postoperative VAS scores in our study were significantly less in the gabapentin and pregabalin groups when compared to the placebo group. Nonetheless, the scores were comparable in the gabapentin and pregabalin groups. This is similar to the results of a study in which patients undergoing lower abdominal and limb surgery under spinal anaesthesia showed significantly lower VAS scores in the pregabalin (300 mg) and gabapentin (900 mg) group when compared to placebo in the first eight hours postoperatively [8]. Patients receiving oral 150 mg pregabalin before the induction of anaesthesia in some other studies showed a significant reduction in VAS scores in the first 24 hours post-surgery [6,9]. In yet another study, preoperative gabapentin produced significantly lower VAS scores, both during rest and movement at one, four, eight, 12, 16, 20, and 24 hours postoperatively in patients undergoing abdominal hysterectomy [10].

The difference in the mean time of postoperative rescue analgesia in the current study was highly significant when the gabapentin group was compared with the placebo group and when the pregabalin group was compared with the placebo group (P value=0.001). This means that the postoperative pain-free period is longer with gabapentin and pregabalin when compared to placebo. However, patients in the pregabalin group had more prolonged pain relief as compared to those in the gabapentin group (P value=0.014). The longer time interval between the administration of spinal anaesthesia to the first dose of analgesic, that is, a longer mean duration of effective analgesia has been observed in some other studies [6,8,11]. The rescue analgesic was given when VAS scores were higher than seven in one of these studies. However, in our study, the rescue analgesic was given when the VAS score was four and above [8].

The total tramadol consumption in our study patients was significantly lower in the pregabalin and gabapentin groups when compared to the placebo group. However, there was no significant difference in the total tramadol consumption between the gabapentin and pregabalin groups. Gabapentin and pregabalin have been found to reduce the dose of rescue analgesics such as fentanyl and diclofenac by 36%-41% in some other studies on patients undergoing surgeries such as abdominal hysterectomy, laparoscopic hysterectomy, and laparoscopic cholecystectomy [12,13].

The level of sedation in the current study was higher in the pregabalin group and gabapentin group patients compared to the placebo group up to six hours in the postoperative period. The sedation scores were similar in the pregabalin and gabapentin group patients. However, this observation is different from that of other authors. In a randomised controlled trial on 90 patients undergoing hysterectomy, 300 mg pregabalin was compared with 900 mg gabapentin, and the incidence of somnolence was found to be 33% in the gabapentin group and 40% in the pregabalin group compared to 3.3% in the control group [4]. There is varied evidence from some other studies as well, wherein both gabapentin and pregabalin were found to be associated with high sedation scores [8,12]. Nevertheless, the sedation that accompanies the analgesic effect of pregabalin and gabapentin may be beneficial because the onset of sedation indicates the reduction of anxiety. Perioperative anxiety leads to a surge of catecholamines due to the stress response leading to tachycardia, hypertension, and haemodynamic instability.

Gabapentin and pregabalin have been used by researchers in doses in the range of 300 mg to 1200 mg and 75-300 mg respectively. We decided on the dose of pregabalin and gabapentin as 300 mg and 600 mg, respectively, after analysing the methodology and results of other studies with regard to drug action, adverse effects, and safety [3].

The incidence of side effects was not significant between the groups in the current study, though dizziness was observed in two patients each in the gabapentin and pregabalin groups. Nevertheless, the authors of an editorial have cautioned about the perioperative use of gabapentinoids. They have said that the last few years have anticipated the increasing evidence of the harmful effects of the perioperative use of gabapentinoids [14,15]. Nonetheless, their perioperative use is now on the decline due to their side effects such as postoperative dizziness, ataxia, visual disturbances, sedation, and respiratory depression.

The findings of our study imply that oral gabapentin 600 mg/pregabalin 300 mg when administered one hour before laparoscopic cholecystectomy produces good postoperative analgesia and that their analgesic efficacy is similar. Both produce sedation of a similar level up to six hours in the postoperative period. The sedation and the occurrence of side effects such as dizziness, ataxia, and vomiting can impact patient outcomes and recovery, especially in this era of enhanced recovery after surgery. However, significant side effects were not observed in our study cases. Gabapentin and pregabalin can be thus included in perioperative multimodal analgesia regimens.

The strength of our study lies in our study design. This was a randomised double-blind controlled study. Moreover, the study results can prove to be clinically useful in a commonly encountered surgical population, viz., laparoscopic cholecystectomy.

Our study is associated with a few limitations. The age of the patients who participated in our study was limited to the non-geriatric range. Moreover, laparoscopic surgery is associated with a significant stress response. However, though the study was conducted in laparoscopic cholecystectomy patients, observations related to the effect of gabapentin and pregabalin in reducing the stress response were not recorded. The vomiting that occurred in two cases in the gabapentin group could have been due to nitrous oxide, and this becomes a confounding factor. Moreover, there is the potential risk of observer bias in the assessment of the occurrence of dizziness. Most of the patients were sedated postoperatively and, hence, their complaints of dizziness were assessed subjectively. Further studies can be conducted on the effect of preoperative gabapentin and pregabalin in the prevention of chronic postsurgical pain.

## Conclusions

Gabapentin and pregabalin are associated with lower rescue analgesic consumption, higher sedation, and a longer postoperative pain-free period than placebo in patients undergoing laparoscopic cholecystectomy. Moreover, pregabalin is associated with significantly lower total rescue analgesic consumption than gabapentin. In conclusion, a single preoperative dose of pregabalin 300 mg or gabapentin 600 mg can be used to provide effective preemptive analgesia in patients undergoing laparoscopic cholecystectomy.

## References

[1] Apfelbaum JL, Chen C, Mehta SS, Gan TJ (2003). Postoperative pain experience: results from a national survey suggest postoperative pain continues to be undermanaged. Anesth Analg.

[2] Raja SN, Carr DB, Cohen M (2020). The revised International Association for the Study of Pain definition of pain: concepts, challenges, and compromises. Pain.

[3] Chang CY, Challa CK, Shah J, Eloy JD (2014). Gabapentin in acute postoperative pain management. Biomed Res Int.

[4] Ghai A, Gupta M, Hooda S, Singla D, Wadhera R (2011). A randomized controlled trial to compare pregabalin with gabapentin for postoperative pain in abdominal hysterectomy. Saudi J Anaesth.

[5] Balaban F, Yağar S, Özgök A, Koç M, Güllapoğlu H (2012). A randomized, placebo-controlled study of pregabalin for postoperative pain intensity after laparoscopic cholecystectomy. J Clin Anesth.

[6] Mishra R, Tripathi M, Chandola HC (2016). Comparative clinical study of gabapentin and pregabalin for postoperative analgesia in laparoscopic cholecystectomy. Anesth Essays Res.

[7] Gill M, Green SM, Krauss B (2003). A study of the bispectral index monitor during procedural sedation and analgesia in the emergency department. Ann Emerg Med.

[8] Rajendran I, Basavareddy A, Meher BR, Srinivasan S (2014). Prospective, randomised, double blinded controlled trial of gabapentin and pregabalin as pre-emptive analgesia in patients undergoing lower abdominal and limb surgery under spinal anaesthesia. Indian Journal of Pain.

[9] Agarwal A, Gautam S, Gupta D, Agarwal S, Singh PK, Singh U (2008). Evaluation of a single preoperative dose of pregabalin for attenuation of postoperative pain after laparoscopic cholecystectomy. Br J Anaesth.

[10] Turan A, Karamanlioğlu B, Memiş D, Hamamcioglu MK, Tükenmez B, Pamukçu Z, Kurt I (2004). Analgesic effects of gabapentin after spinal surgery. Anesthesiology.

[11] Bafna U, Rajarajeshwaran K, Khandelwal M, Verma AP (2014). A comparison of effect of preemptive use of oral gabapentin and pregabalin for acute post-operative pain after surgery under spinal anesthesia. J Anaesthesiol Clin Pharmacol.

[12] Pandey CK, Priye S, Singh S, Singh U, Singh RB, Singh PK (2004). Preemptive use of gabapentin significantly decreases postoperative pain and rescue analgesic requirements in laparoscopic cholecystectomy. Can J Anaesth.

[13] Rorarius MG, Mennander S, Suominen P (2004). Gabapentin for the prevention of postoperative pain after vaginal hysterectomy. Pain.

[14] Bajwa SJ (2021). Dexmedetomidine and Ketamine - Comrades on an eternal journey!. Indian J Anaesth.

[15] Kharasch ED, Clark JD, Kheterpal S (2020). Perioperative gabapentinoids: deflating the bubble. Anesthesiology.

